# Supplementary material for: Nodal lymphangiogenesis and immunophenotypic variations of sinus endothelium in sentinel and non-sentinel lymph nodes of invasive breast carcinoma
Source: PLoS One. 2023 Jan 24;18(1):e0280936. doi: 10.1371/journal.pone.0280936 (PMC9873157; doi:10.1371/journal.pone.0280936)
Supplement: S1 Table — LN: lymph node, ITC: isolated tumor cells, SLN: sentinel lymph node, ILC: invasive lobular carcinoma, NST: invasive carcinoma of no special type, tubular: tubular carcinoma, NSLN: non sentinel lymph node, NA: not applicable. (DOCX) [file pone.0280936.s001.docx]

| **LN metastasis**  **Tumor type** | **Negative**  **n (%)** | **ITC**  **n (%)** | **micrometastasis**  **(> 0.2 mm), n (%)** | **macrometastasis**  **(> 0.2 cm), n (%)** | **p-value** |
| --- | --- | --- | --- | --- | --- |
| **Cases of SLN** | n=60 | n=3 | n=16 | n=16 | 0.449 |
| NST | 48 (80.0%) | 1 (33.3%) | 13 (81.2%) | 13 (81.2%) |  |
| ILC | 10 (16.7%) | 2 (66.7%) | 3 (18.8%) | 3 (18.8%) |  |
| tubular | 2 (3.33%) | 0 (0.00%) | 0 (0.00%) | 0 (0.00%) |  |
| **Cases of NSLN** | n=32 |  | n=3 | n=5 | 0.882 |
| NST | 21 (65.6%) | NA | 3 (100%) | 4 (80.0%) |  |
| ILC | 9 (28.1%) | NA | 0 (0.00%) | 1 (20.0%) |  |
| tubular | 2 (6.25%) | NA | 0 (0.00%) | 0 (0.00%) |  |
