# Supplementary material for: Nodal lymphangiogenesis and immunophenotypic variations of sinus endothelium in sentinel and non-sentinel lymph nodes of invasive breast carcinoma
Source: PLoS One. 2023 Jan 24;18(1):e0280936. doi: 10.1371/journal.pone.0280936 (PMC9873157; doi:10.1371/journal.pone.0280936)
Supplement: S2 Table — CC: Chalkley count, SLN: sentinel lymph node, ILC: invasive lobular carcinoma, NST: invasive carcinoma of no special type, NSLN: non sentinel lymph node. (DOCX) [file pone.0280936.s002.docx]

| **CC category**  **Tumor type** | **low** | **high** | **p.overall** |
| --- | --- | --- | --- |
| **Cases of SLN** | n=10 | n=10 | 1.000 |
| ILC | 1 (10.0%) | 2 (20.0%) |  |
| NST | 9 (90.0%) | 8 (80.0%) |  |
| **Cases of NSLN** | n=3 | n=2 | 0.400 |
| ILC | 0 (0.00%) | 1 (50.0%) |  |
| NST | 3 (100%) | 1 (50.0%) |  |
