# Supplementary material for: Nodal lymphangiogenesis and immunophenotypic variations of sinus endothelium in sentinel and non-sentinel lymph nodes of invasive breast carcinoma
Source: PLoS One. 2023 Jan 24;18(1):e0280936. doi: 10.1371/journal.pone.0280936 (PMC9873157; doi:10.1371/journal.pone.0280936)
Supplement: S3 Table — NST: invasive carcinoma of no special type, tubular: tubular carcinoma, LN: lymph node, ITC: isolated tumor cells. (DOCX) [file pone.0280936.s003.docx]

| **Proportion of D2-40 stained sinus, categorized** | **0 %**  n=24 | **< 10%**  n=23 | **10 - 50%**  n=32 | **51 -80%**  n=9 | **>80%**  n=7 | **p.overall** |
| --- | --- | --- | --- | --- | --- | --- |
| **Grading** | | | | | | 0.621 |
| **G1** | 1 (4.17%) | 3 (13.0%) | 1 (3.12%) | 0 (0.00%) | 0 (0.00%) |  |
| **G2** | 22 (91.7%) | 20 (87.0%) | 28 (87.5%) | 8 (88.9%) | 7 (100%) |  |
| **G3** | 1 (4.17%) | 0 (0.00%) | 3 (9.38%) | 1 (11.1%) | 0 (0.00%) |  |
| **Size of primary tumor (in mm)** | 13.8 (4.62) | 13.7 (6.70) | 14.8 (8.23) | 16.7 (6.87) | 16.1 (4.81) | 0.727 |
| **Tumor type** | | | | | | 0.600 |
| **invasive lobular** | 3 (12.5%) | 5 (21.7%) | 6 (18.8%) | 2 (22.2%) | 2 (28.6%) |  |
| **invasive NST** | 21 (87.5%) | 16 (69.6%) | 26 (81.2%) | 7 (77.8%) | 5 (71.4%) |  |
| **tubular** | 0 (0.00%) | 2 (8.70%) | 0 (0.00%) | 0 (0.00%) | 0 (0.00%) |  |
| **pT stage** | | | | | | . |
| **pT1a** | 0 (0.00%) | 2 (8.70%) | 0 (0.00%) | 0 (0.00%) | 0 (0.00%) |  |
| **pT1b** | 5 (20.8%) | 5 (21.7%) | 10 (31.2%) | 3 (33.3%) | 0 (0.00%) |  |
| **pT1c** | 18 (75.0%) | 13 (56.5%) | 16 (50.0%) | 3 (33.3%) | 5 (71.4%) |  |
| **pT2** | 1 (4.17%) | 3 (13.0%) | 5 (15.6%) | 3 (33.3%) | 2 (28.6%) |  |
| **pT3** | 0 (0.00%) | 0 (0.00%) | 1 (3.12%) | 0 (0.00%) | 0 (0.00%) |  |
| **pN stage** | | | | | | . |
| **pN0** | 17 (70.8%) | 16 (69.6%) | 23 (71.9%) | 4 (44.4%) | 5 (71.4%) |  |
| **pN1mi** | 4 (16.7%) | 2 (8.70%) | 4 (12.5%) | 2 (22.2%) | 0 (0.00%) |  |
| **pN1a** | 3 (12.5%) | 3 (13.0%) | 3 (9.38%) | 2 (22.2%) | 1 (14.3%) |  |
| **pN1b** | 0 (0.00%) | 0 (0.00%) | 1 (3.12%) | 0 (0.00%) | 0 (0.00%) |  |
| **pN1c** | 0 (0.00%) | 0 (0.00%) | 0 (0.00%) | 0 (0.00%) | 1 (14.3%) |  |
| **pN2a** | 0 (0.00%) | 1 (4.35%) | 0 (0.00%) | 1 (11.1%) | 0 (0.00%) |  |
| **pN3a** | 0 (0.00%) | 1 (4.35%) | 1 (3.12%) | 0 (0.00%) | 0 (0.00%) |  |
| **Estrogen receptor status** | | | | | | 0.291 |
| **negative** | 5 (20.8%) | 1 (4.35%) | 5 (16.1%) | 0 (0.00%) | 0 (0.00%) |  |
| **positive** | 19 (79.2%) | 22 (95.7%) | 26 (83.9%) | 9 (100%) | 7 (100%) |  |
| **Progesterone receptor status** | | | | | | 0.133 |
| **negative** | 8 (33.3%) | 2 (8.70%) | 9 (29.0%) | 2 (22.2%) | 0 (0.00%) |  |
| **positive** | 16 (66.7%) | 21 (91.3%) | 22 (71.0%) | 7 (77.8%) | 7 (100%) |  |
| **Her2 status** | | | | | | 0.437 |
| **negative** | 21 (87.5%) | 19 (82.6%) | 21 (67.7%) | 8 (88.9%) | 6 (85.7%) |  |
| **positive** | 3 (12.5%) | 4 (17.4%) | 10 (32.3%) | 1 (11.1%) | 1 (14.3%) |  |
| **Age at surgery (in years)** | 53.9 (12.4) | 53.0 (11.8) | 56.5 (10.6) | 55.1 (13.0) | 60.6 (14.0) | 0.571 |
| **Type of LN metastasis** | | | | | | 0.226 |
| **negative** | 17 (70.8%) | 14 (60.9%) | 22 (68.8%) | 3 (33.3%) | 4 (57.1%) |  |
| **ITC** | 0 (0.00%) | 0 (0.00%) | 2 (6.25%) | 0 (0.00%) | 1 (14.3%) |  |
| **micrometastasis** | 5 (20.8%) | 5 (21.7%) | 3 (9.38%) | 3 (33.3%) | 0 (0.00%) |  |
| **macrometastasis** | 2 (8.33%) | 4 (17.4%) | 5 (15.6%) | 3 (33.3%) | 2 (28.6%) |  |
| **Size of LN metastasis (in mm)** | 2.42 (3.12) | 8.50 (4.15) | 7.35 (4.60) | 5.33 (4.51) | 8.25 (2.47) | 0.249 |
