# Supplementary figures and images for: Nodal lymphangiogenesis and immunophenotypic variations of sinus endothelium in sentinel and non-sentinel lymph nodes of invasive breast carcinoma
Source: PLoS One. 2023 Jan 24;18(1):e0280936. doi: 10.1371/journal.pone.0280936 (PMC9873157; doi:10.1371/journal.pone.0280936)

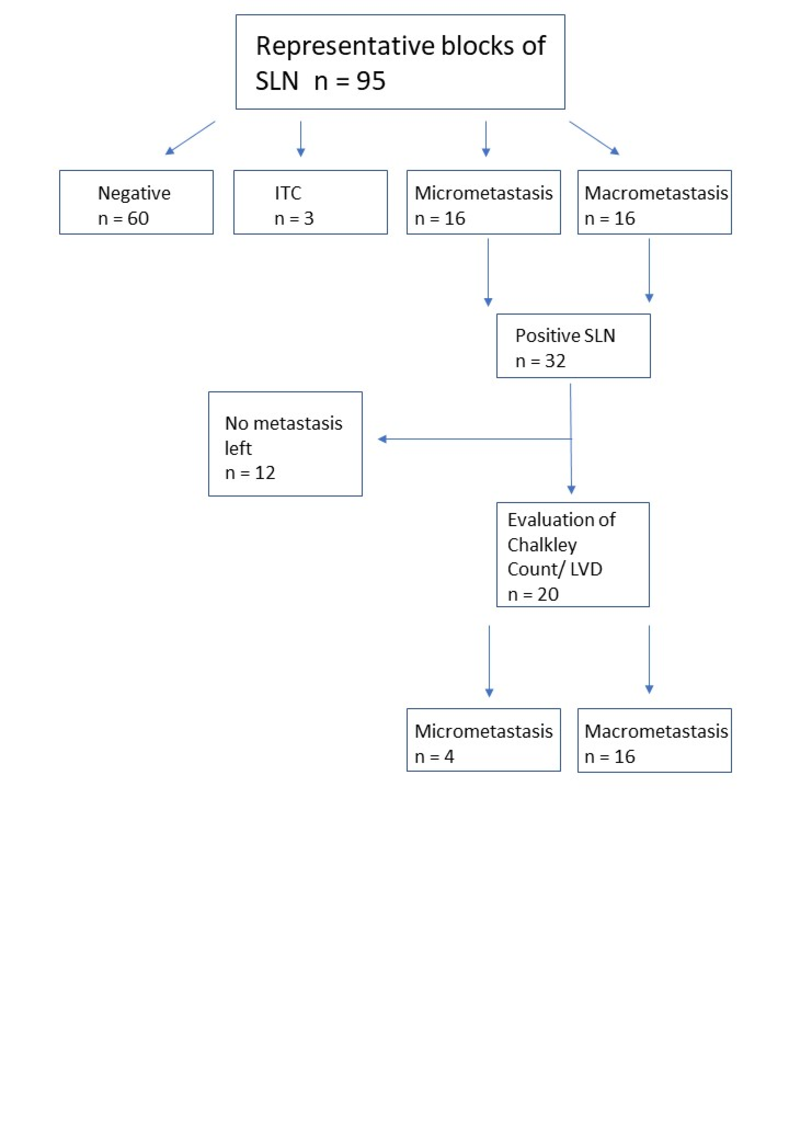

Supplement: S1 Fig — ITC: isolated tumor cells, LVD: lymphatic vessel density. (TIF) [file pone.0280936.s004.tif]

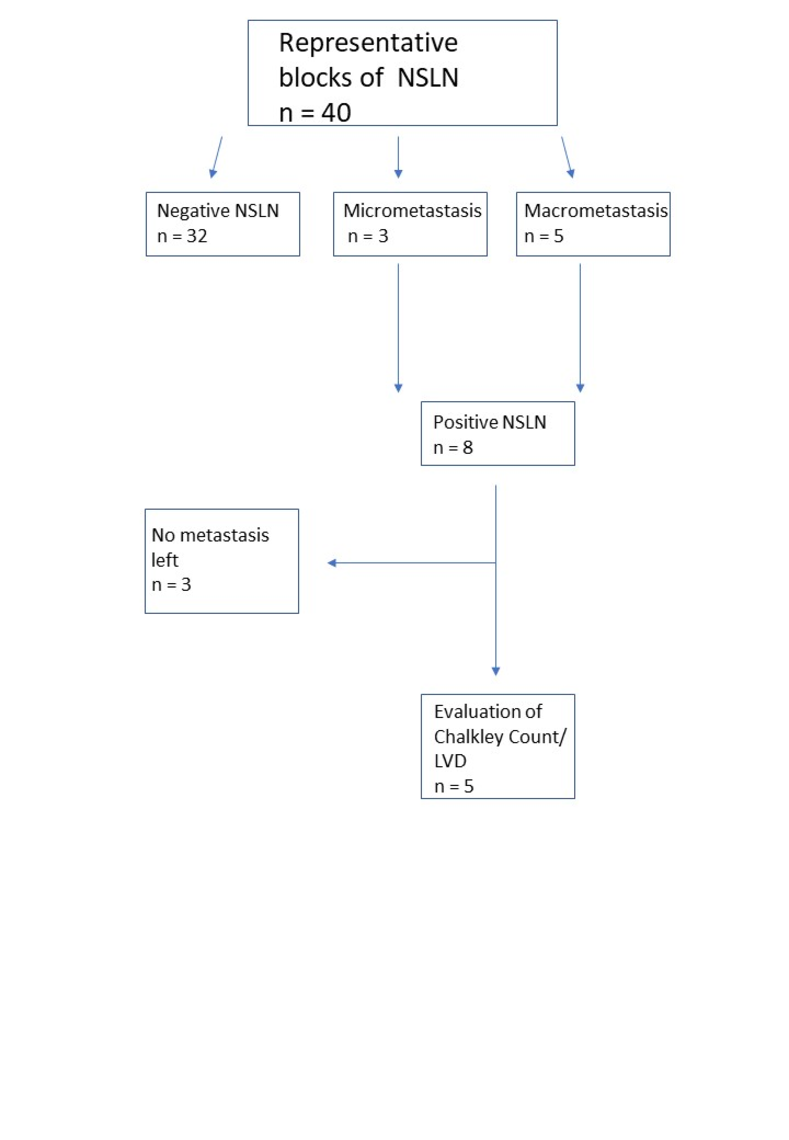

Supplement: S2 Fig — LVD: lymphatic vessel density. (TIF) [file pone.0280936.s005.tif]

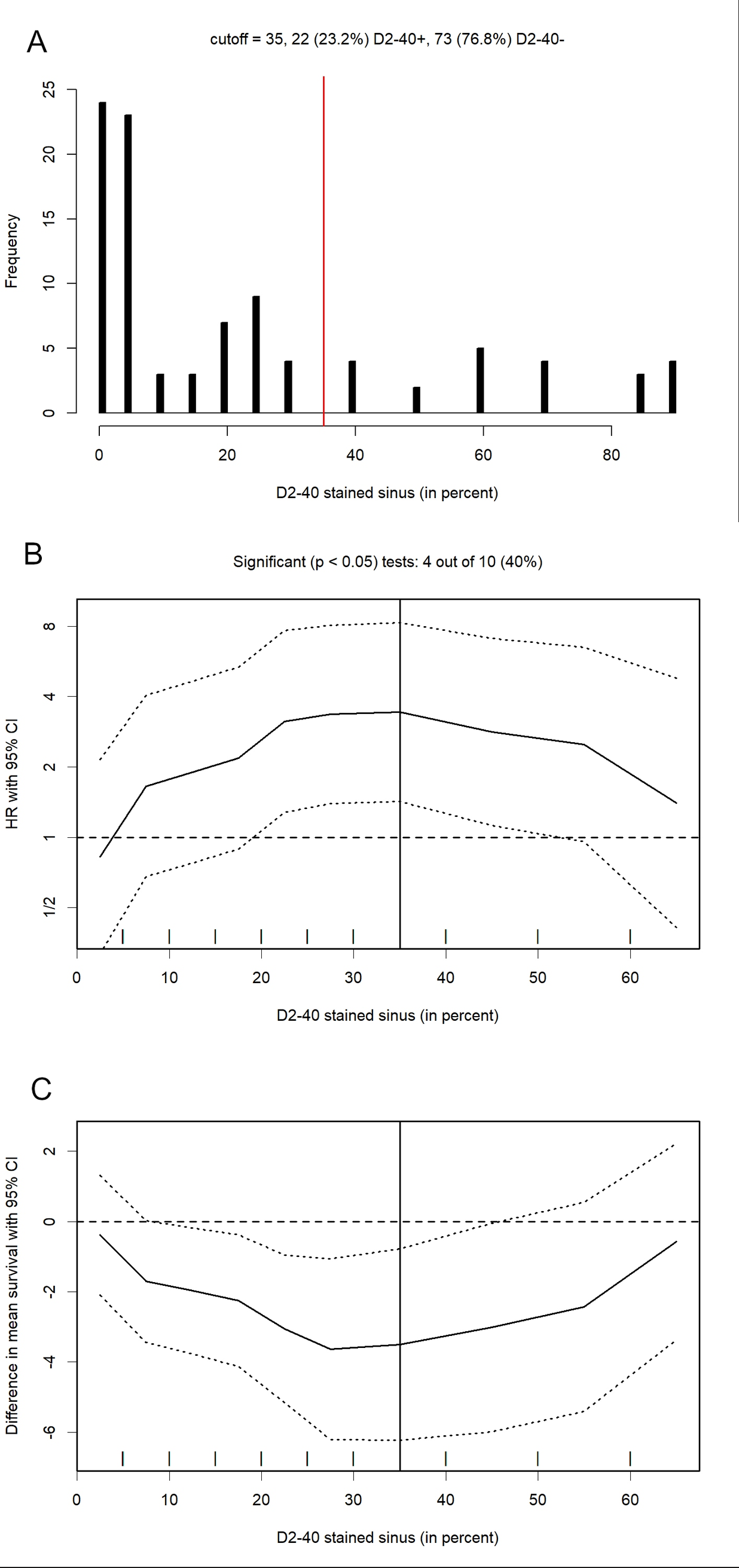

Supplement: S3 Fig — (A) Histogram of proportion of D2-40 stained sinus in the 95 patients analyzed. The vertical line designates the optimal cutoff derived from the model. (B) For each possible cutoff, proportion of D2-40 stained sinus is correlated with overall survival. The hazard ration (HR) including 95% confidence interval (CI) is plotted in dependence of the cutoff. A vertical line designates the dichotomization showing the most significant correlation with survival. (C) The mean survival time is estimated in samples where proportion of D2-40 stained sinus is high and low, respectively. The difference of the mean survival times including 95% CI is plotted. (TIF) [file pone.0280936.s006.tif]
