# Supplementary material for: Nodal lymphangiogenesis and immunophenotypic variations of sinus endothelium in sentinel and non-sentinel lymph nodes of invasive breast carcinoma
Source: PLoS One. 2023 Jan 24;18(1):e0280936. doi: 10.1371/journal.pone.0280936 (PMC9873157; doi:10.1371/journal.pone.0280936)
Supplement: Attachment — Submitted filename: 20220628_Response to reviewer_text.docx [file pone.0280936.s007.docx]

Response to reviewer

Reviewer 2

1. The authors need to state the objective of the current study clearly. In their own words “We now aimed to evaluate these findings in a typical patient cohort of a BC center employing the sentinel node biopsy since 2000. An important objective was the feasibility with routine immunohistochemical repertoire,” “Differentiation for tumor types, especially for invasive lobular carcinoma, was also important to us” “A special emphasis was put on the analysis of the subcapsular sinus endothelium and the possible clinical relevance of its podoplanin expression.”

Though they have expressed a general direction of inquiry, the exact objectives of the study need to be laid out more clearly in unambiguous statements.

We thank the reviewer for demanding more clearly stated objectives and have adjusted the final paragraph of the introduction accordingly.

2. Methodology

a. Why were archived specimens from 16-20 years ago chosen for analysis and not recent ones?

We have chosen archived specimens due to several reasons: First, the specimens are part of a clinical cohort employing the SLN technique relatively early and compiling some clinically oriented data within this study cohort. Second, for significant conclusions regarding prognosis, a cohort with long term follow-up regarding survival is indispensable.

b.The authors need to mention in the statistical methodology section, what was the null hypothesis. What was the expected percentage staining in NSLN nodes, and the expected difference between SLN and NSLN in terms of lymphangiogenesis within metastases and in the subcapsular sinus?

We thank the reviewer for requiring clarification with regard to the null hypothesis. We have adjusted the last paragraph of the materials and methods section accordingly. With respect to the expected percentage of D2-40 staining in subcapsular sinus of SLN/ NSLN, the presented study has explorative character. With respect to expected difference between SLN and NSLN in terms of lymphangiogenesis, a previous study detected positive NSLN in 37 out of 65 patients with positive SLN in BC [1].

c.What was the rationale for choosing a multivariate model with only the following as independent variables? age at surgery, pN stage, tumor size. Why were other prognostic variables not included (eg. T stage, ER, PR, HER2 status, grade)

For multivariate models, care must be taken in balancing the variable of interest with the relevant, known prognostic variables with respect to both number of variables and number of categories within the variables.

In previous studies of our group, the most constant statistically relevant variables regarding overall survival were T/N stage and age at surgery. For age as continuous variable, we used the standardized tool “Cutoff Finder” to determine 63.5 years as best cutoff point for OS. Since T stage is a parameter of tumor size and the predominant pT stage was pT1, we determined 17.5 mm as best cutoff point for tumor size accordingly.

In our experience, grading is not necessarily equivalent to T/N stage as prognostic parameter in smaller study cohorts. Regarding hormone receptor and HER2 status, data quality is not comparable to large multicenter studies, e.g., absolute percentage is not available for hormone receptor status.

We have also repeated multivariate analysis for age (dichotomized), pT stage, pN stage and D2-40/ podoplanin expression (dichotomized) with virtually the same result. We have exchanged Figure 7 with this version.

d.The authors have not taken into account the treatment patient received when assessing factors influencing survival. The receptor status and treatment received in terms of antiHER2 directed therapy, anthracycline and taxane are very important prognostic factors for survival which cannot be ignored. They should either mention these as a major drawback of the survival analysis or give data regarding the above.

We thank the reviewer for his important remark. Data on treatment is not available respectively data quality not appropriately controlled for in this study cohort (e.g. adjuvant treatment agent, dose and duration), the proportion of HER2 positive cases is 20% meeting the expected share. We have included a statement indicating this limitation in the discussion (second to last paragraph).

e.Wherever continuous variables are compared using Pearson or Spearman, the correlation coefficient should be mentioned alongside the p-value in the text for the readers to understand the strength of association.

The correlation coefficients are mentioned in the original manuscript (Results, page 10, paragraph between figure captions 1 and 2), p values have been added for completeness.

3.“In contrast to other studies, we performed a precise, yet straightforward analysis and evaluation easily employable in the daily routine of clinical pathology”.

The authors need to elucidate the clinical importance of demonstrating lymphangiogenesis within LN with metastases. The KM curves for OS and the multivariate analysis are encouraging for podoplanin as a biomarker which may predict survival. This requires prospective validation, however.

We have included the need for prospective validation in the final sentence of the discussion, in addition to our already tentative conclusions regarding the potential of podoplanin as biomarker.

4.The authors need to clarify the flowchart (STROBE) of the number of specimens studied with respect to SLN, NSLN and all cases. The following statements mention N but it is not clear to the reader. Perhaps the flow of N cases starting from archived specimens can be mentioned in the results or methodology, if not graphically represented.

-“Representative paraffin blocks of 75 SLN and/or non-SLN (NSLN) from n= 95 patients were recruited”

-“All cases of SLN and NSLN without micro- or macrometastasis in the present study, comprising 63 cases”

-“Among 95 SLN overall 134 investigated, 32 harbored metastases”

-“In 40 cases, additional NSLN could be investigated”

-“Established lymph node metastases in 20 SLN and five NSLN were available for further immunohistochemical analysis of newly formed D2-40 positive lymphatic vessels”

We have followed the reviewer’s advice and included flowcharts as supplementary figures.

5. What was the association between the proportion of D2-40 stained subcapsular sinus in NSLN and overall survival?

There is no statistically significant association between the proportion of D2-40 stained subcapsular sinus in NSLN and overall survival. For legibility, only significant results have been reported.

6. The authors need to elucidate why the concordance between staining intensity and proportion of stained sinus is clinically important.

It is yet too early for conclusions regarding the clinical significance of the concordance between staining intensity and proportion, we have added a statement in the second to last paragraph of the discussion.

7. The authors could provide some data on the difference in D2-40 staining between breast cancer subtypes(ER, PR, HER2) if available. In the supplementary table, subcapsular sinus staining across subtypes is presented, however the association between subtype and lymphangiogenesis in metastasis is not mentioned.

Regarding hormone receptor and HER2 status (and thus molecular breast cancer subgroups), data quality is not comparable to large multicenter studies, e.g., absolute percentage is not available for hormone receptor status.

Tables S1 and S2 include data on tumor type related to status and size of lymph node metastasis (table S1) and Chalkley count category (table S2) for both SLN and NSLN, with Chalkley count measuring the lymphangiogenesis in metastasis.

P2 Line 22

Suggestion- change to “(podoplanin) “expression on” lymphatic vessels and the subcapsular sinus.”

P2 Line 24

Suggestion- Data was explored “with respect to”

We have corrected the abstract according to the suggestions of the reviewer, with a modification in line 22. We have made additional adjustments to meet the requested limit of words within the abstract.

We have also corrected mistakes in the formatting of authors (line 5) and deleted the current address of the second author due to a return to her previous institution in the meantime.

Table 1

When mean is used as a measure of central tendency, the standard deviation is mentioned instead of range. This is applicable if the variable is distributed normally. This is unlikely to be the case with such small numbers, hence the better statistic to represent central tendency is median and IQR in most of the variables unless normal distribution is confirmed with a histogram or other methods.

Table 1 contains mainly descriptive characterization of the study population without statistics associated. For the continuous variables (age and tumor size) normal distribution can be confirmed. As mentioned in the table heading, mean with range in brackets is given for continuous variables. In our opinion, this approach represents the characteristics of our study population best. We have complemented the heading and parameters age and size for clarification.

In this table it would be better to mention the number of patients who were triple-negative(ER, PR and HER2neu negative) separately. This number cannot be obtained from the table.

The number of triple negative cases has been added in Table 1.

Figure 6

Please provide population at risk table along with KM curve if possible

Population at risk table has been added in Figure 6.

Additional changes have been made to the manuscript with respect to the current address of the second author and citation for R packages used has been updated.

References:

**1**.	Van den Eynden, Gert G, Vandenberghe MK, van Dam P-JH, Colpaert CG, van Dam P, Dirix LY, et al. Increased sentinel lymph node lymphangiogenesis is associated with nonsentinel axillary lymph node involvement in breast cancer patients with a positive sentinel node. Clin Cancer Res. 2007; 13:5391–7. doi: 10.1158/1078-0432.CCR-07-1230 PMID: 17875768.
